# Crystal structure of tri­methyl­ammonium 5-(2,4-di­nitro­phen­yl)-1,3-dimethyl-2,6-dioxo-1,2,3,6-tetra­hydro­pyrimidin-4-olate

**DOI:** 10.1107/S1600536814019977

**Published:** 2014-09-13

**Authors:** Sridevi Gunaseelan, Kalaivani Doraisamyraja

**Affiliations:** a PG and Research Department of Chemistry, Seethalakshmi Ramaswami College, Tiruchirappalli 620 002, Tamil Nadu, India

**Keywords:** crystal structure, barbiturates, biological activity, tri­methyl­ammonium salt, tetra­hydro­pyrimidin-4-olate salt, anionic σ-complexes

## Abstract

The asymmetric unit of the title mol­ecular salt, C_3_H_10_N^+^·C_12_H_9_N_4_O_7_^−^ [alternative name: tri­methyl­ammonium 5-(2,4-di­nitro­phen­yl)-1,3-dimethyl barbiturate], contains one anion and two half-occupancy cations. The cations are disordered about inversion centres. The tetra­hydro­pyrimidine ring is essentially planar [maximum deviation = 0.007 (2) Å] and forms a dihedral angle of 41.12 (6)° with the plane of the benzene ring. In the crystal, N—H⋯O hydrogen bonds link the cations to the anions.

## Related literature   

For the biological activity of barbiturates, see: Hueso *et al.* (2003 ▶); Kalaivani *et al.* (2008 ▶); Tripathi (2009 ▶); Kalaivani & Buvaneswari (2010 ▶). For various types of anionic σ-complexes, see: Terrier (1982 ▶); Gnanadoss & Kalaivani (1985 ▶); Al-Kaysi *et al.* (2005 ▶); For barbiturates as carbon-bonded σ-complexes, see: Kalaivani & Malarvizhi (2009 ▶); Buvaneswari & Kalaivani (2011 ▶); Kalaivani *et al.* (2012 ▶); Babykala & Kalaivani (2012 ▶, 2013 ▶); Sridevi & Kalaivani (2012 ▶); Rajamani & Kalaivani (2012 ▶). For the crystal structure of a related barbiturate, see: Mangaiyarkarasi & Kalaivani (2013 ▶)
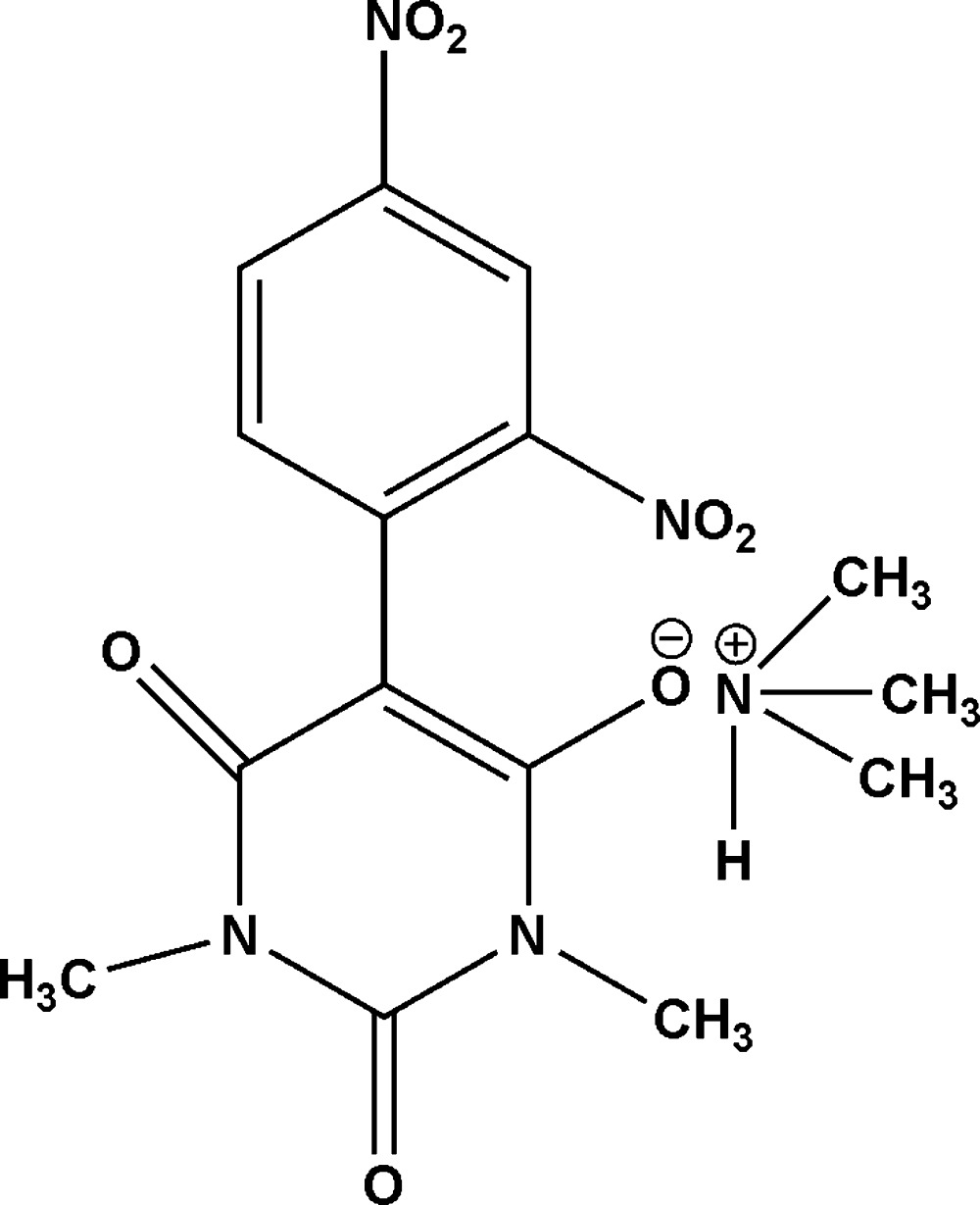


## Experimental   

### Crystal data  

C_3_H_10_N^+^·C_12_H_9_N_4_O_7_^−^
*M* *_r_* = 381.35
Triclinic, 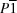

*a* = 9.8417 (5) Å
*b* = 9.9474 (6) Å
*c* = 10.4241 (5) Å
α = 103.454 (2)°
β = 106.479 (2)°
γ = 100.856 (2)°
*V* = 915.66 (9) Å^3^
*Z* = 2
Mo *K*α radiation
μ = 0.11 mm^−1^
*T* = 293 K
0.35 × 0.35 × 0.30 mm

### Data collection  

Bruker Kappa APEXII CCD diffractometer
Absorption correction: multi-scan (*SADABS*; Bruker, 2004 ▶) *T*_min_ = 9536, *T*_max_ = 9865
16319 measured reflections
4271 independent reflections
2922 reflections with *I* > 2σ(*I*)
*R*_int_ = 0.026

### Refinement  

*R*[*F* ^2^ > 2σ(*F* ^2^)] = 0.051
*wR*(*F* ^2^) = 0.164
*S* = 0.99
4271 reflections
341 parameters
76 restraints
H atoms treated by a mixture of independent and constrained refinement
Δρ_max_ = 0.23 e Å^−3^
Δρ_min_ = −0.20 e Å^−3^

### 

Data collection: *APEX2* (Bruker, 2004 ▶); cell refinement: *APEX2* and *SAINT* (Bruker, 2004 ▶); data reduction: *SAINT* and *XPREP* (Bruker, 2004 ▶); program(s) used to solve structure: *SIR92* (Altomare *et al.*, 1993 ▶); program(s) used to refine structure: *SHELXL97* (Sheldrick, 2008 ▶); molecular graphics: *ORTEP-3 for Windows* (Farrugia, 2012 ▶) and *Mercury* (Macrae *et al.*, 2008 ▶); software used to prepare material for publication: *SHELXL97*.

## Supplementary Material

Crystal structure: contains datablock(s) global, I. DOI: 10.1107/S1600536814019977/lh5722sup1.cif

Structure factors: contains datablock(s) I. DOI: 10.1107/S1600536814019977/lh5722Isup2.hkl

Supporting information file. DOI: 10.1107/S1600536814019977/lh5722Isup3.cml

**Fig. 1.**
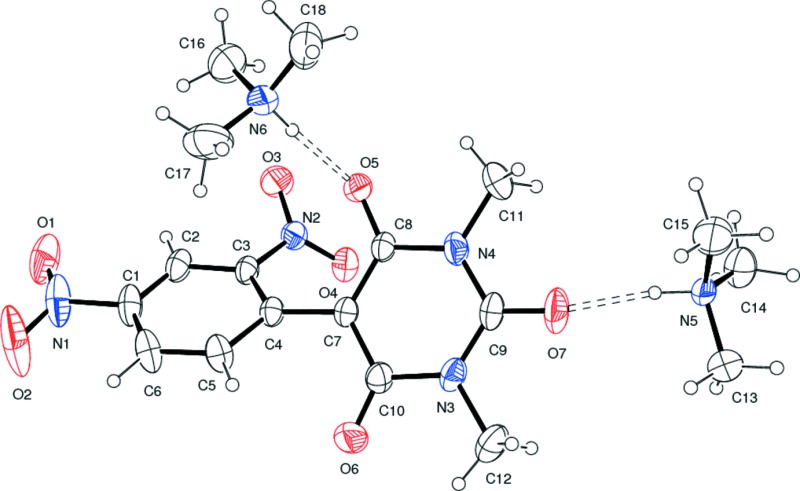
The asymmetric unit of title compound showing 30% probability displacement ellipsoids. The cations are half occupancy.

. DOI: 10.1107/S1600536814019977/lh5722fig1.tif
The asymmetric unit of title compound showing 30% probability displacement ellipsoids. The cations are half occupancy.

CCDC reference: 1022943

Additional supporting information:  crystallographic information; 3D view; checkCIF report

## Figures and Tables

**Table 1:** Hydrogen-bond geometry (Å, °)

*D*—H⋯*A*	*D*—H	H⋯*A*	*D*⋯*A*	*D*—H⋯*A*
N5—H5*A*⋯O7	0.91 (2)	1.80 (2)	2.666 (3)	156 (2)
N6—H6*A*⋯O5	0.92 (2)	1.83 (2)	2.737 (3)	171 (2)

## supplementary crystallographic information

### S1. Comment

Barbiturates are pyrimidine derivatives and most of them have anticonvulsant activity (Hueso *et al.*, 2003; Kalaivani *et al.*, 2008; Tripathi, 2009; Kalaivani & Buvaneswari, 2010). Various different types of anionic sigma complexes such as carbon-bonded, nitrogen-bonded, oxygen-bonded and Spiro-Meisenheimer complexes have been reported by different groups of scientists (Terrier, 1982; Gnanadoss & Kalaivani, 1985; Al-Kaysi *et al.*, 2005). A number of crystalline barbiturates in the form of carbon-bonded sigma complexes have been prepared and reported by our group (Kalaivani & Malarvizhi, 2009; Buvaneswari & Kalaivani, 2011; Kalaivani *et al.*, 2012; Babykala & Kalaivani, 2012; Sridevi & Kalaivani, 2012; Rajamani & Kalaivani, 2012; Babykala & Kalaivani, 2013). The asymmetric unit of a related barbiturate prepared from 1-chloro-2,4-dinitrobenzene (DNCB) and barbituric acid in the presence of trimethylamine (Mangaiyarkarasi & Kalaivani, 2013) comprises of two cations and two anions. However, in the present investigation, the asymmetric unit of the molecular salt is comprised of one 5-(2,4-dinitrophenyl)-1,3-dimethylbarbiturate anion and two half occupancy trimethylammonium cations. The molecular structure of the title compound is shown in Fig. 1. The cations lie on inversion centres and hence are disordered. In the crystal, N—H···O hydrogen bonds exist between the protonated nitrogen atoms of the cations and oxygen atoms of the carbonyl groups of anions. The tetrahydropyrimidine ring is essentially planar (maximum deviation = 0.007 (2) Å for N3) and forms a dihedral angle of 41.12 (6)Å with the benzene ring. The nitro group which *para* with respect to the junction of two rings forms a dihedral of -5.7 (2)° with the benzene ring and may be involved to a greater extent in electron delocalization with the benzene than the nitro group *ortho* to the junction of the two rings which forms a dihedral angle of 38.8 (2)° with the benzene ring.

### S2. Experimental

Analytical grade 1-chloro-2,4-dinitrobenzene (2.02 g,0.01 mol) was dissolved in 20 ml of absolute alcohol. 1,3-Dimethylbarbituric acid (1.56 g,0.01 mol) was also dissolved in 30 ml of absolute alcohol separately. These two solutions were then mixed. To this mixture, 4 ml of trimethylamine (0.03 mol) was added and shaken well for 5–6 hrs. The slightly turbid solution obtained was filtered and kept as such at 298K. After a period of three weeks, dark shiny maroon red coloured crystals of the title salt crystallized out from the solution. The crystals were filtered, powdered well using agate mortar and washed with 30 ml of dry ether. The dry solid of the title compound obtained was quickly washed with 1 ml of absolute alcohol to remove unreacted reactants and finally with 25 ml of dry ether. The pure powder was recrystllized from hot etanol (Yield: 80%; m.pt; 513 K). Good quality single crystals, suitable for X-ray diffraction studies, were obtained by slow evaporation of a solution of the title compound in ethanol at room temperature. The crystals obtained were non-hygroscopic and extraordinarily stable at room temperature. Solubility at 298 K: 2.78 g/100 mL(water); 4.58 g/100 mL(Ethanol); 17.78 g/100 mL(DMSO).

### S3. Refinement

The hydrogen atoms boned to the N atoms of the cation were located in a difference Fourier map and refined isotropically with U_iso_(H) = 1.2U_eq_(N) and restrained to N—H = 0.91 (2)Å. Hydrogen atoms on methyl groups of the trimethyl ammonium cation were located in a difference Fourier map and refined with fixed isotropic displacement parameters with U_iso_(H) = 1.5U_eq_(C). The C—H and H···H distances were restrained to 0.96 (2)Å and 1.568 (2)Å respectively. All the other hydrogen atoms were geometrically constrained and allowed to ride on their parent atoms. The cations are disordered across inversion centres. There are two cation moieties in the asymmetric unit each with 0.5 site occupancy. The anisotropic displacement parameters of the disordered atoms were restrained with an effective standard deviation of 0.02. The N—C and C···C distances were restrained to 1.45 (2) Å and 2.36 (2) Å respectively.

### Crystal data

**Table:**

C_3_H_10_N^+^·C_12_H_9_N_4_O_7_^−^	*Z* = 2
*M**_r_* = 381.35	*F*(000) = 400
Triclinic, *P*1	*D*_x_ = 1.383 Mg m^−^^3^
*a* = 9.8417 (5) Å	Mo *K*α radiation, λ = 0.71073 Å
*b* = 9.9474 (6) Å	Cell parameters from 5386 reflections
*c* = 10.4241 (5) Å	θ = 2.1–27.5°
α = 103.454 (2)°	µ = 0.11 mm^−^^1^
β = 106.479 (2)°	*T* = 293 K
γ = 100.856 (2)°	Block, red
*V* = 915.66 (9) Å^3^	0.35 × 0.35 × 0.30 mm

### Data collection

**Table:**

Bruker Kappa APEXII CCD diffractometer	2922 reflections with *I* > 2σ(*I*)
Radiation source: fine-focus sealed tube	*R*_int_ = 0.026
ω and φ scan	θ_max_ = 27.7°, θ_min_ = 2.1°
Absorption correction: multi-scan (*SADABS*; Bruker, 2004)	*h* = −12→12
*T*_min_ = 9536, *T*_max_ = 9865	*k* = −12→12
16319 measured reflections	*l* = −13→13
4271 independent reflections	

### Refinement

**Table:**

Refinement on *F*^2^	Hydrogen site location: mixed
Least-squares matrix: full	H atoms treated by a mixture of independent and constrained refinement
*R*[*F*^2^ > 2σ(*F*^2^)] = 0.051	*w* = 1/[σ^2^(*F*_o_^2^) + (0.0891*P*)^2^ + 0.1912*P*] where *P* = (*F*_o_^2^ + 2*F*_c_^2^)/3
*wR*(*F*^2^) = 0.164	(Δ/σ)_max_ < 0.001
*S* = 0.99	Δρ_max_ = 0.23 e Å^−^^3^
4271 reflections	Δρ_min_ = −0.20 e Å^−^^3^
341 parameters	Extinction correction: *SHELXL97* (Sheldrick, 2008), Fc^*^=kFc[1+0.001xFc^2^λ^3^/sin(2θ)]^-1/4^
76 restraints	Extinction coefficient: 0.020 (4)

### Special details

**Table:**

Geometry. All esds (except the esd in the dihedral angle between two l.s. planes) are estimated using the full covariance matrix. The cell esds are taken into account individually in the estimation of esds in distances, angles and torsion angles; correlations between esds in cell parameters are only used when they are defined by crystal symmetry. An approximate (isotropic) treatment of cell esds is used for estimating esds involving l.s. planes.

### Fractional atomic coordinates and isotropic or equivalent isotropic displacement parameters (Å^2^)

**Table:**

	*x*	*y*	*z*	*U*_iso_*/*U*_eq_	Occ. (<1)
C1	0.3976 (2)	0.7207 (3)	0.07803 (19)	0.0655 (6)	
C2	0.2837 (2)	0.6256 (2)	0.08545 (17)	0.0548 (5)	
H2	0.2434	0.5342	0.0208	0.066*	
C3	0.22965 (18)	0.66915 (18)	0.19261 (16)	0.0440 (4)	
C4	0.29063 (16)	0.80221 (18)	0.29787 (16)	0.0424 (4)	
C5	0.40705 (19)	0.8947 (2)	0.28249 (19)	0.0584 (5)	
H5	0.4508	0.9853	0.3481	0.070*	
C6	0.4590 (2)	0.8561 (3)	0.1734 (2)	0.0712 (6)	
H6	0.5347	0.9208	0.1644	0.085*	
C7	0.24320 (16)	0.84301 (17)	0.41888 (15)	0.0415 (4)	
C8	0.21150 (17)	0.74090 (18)	0.48671 (16)	0.0435 (4)	
C9	0.1589 (2)	0.9213 (2)	0.65492 (18)	0.0560 (5)	
C10	0.23385 (19)	0.98455 (19)	0.46730 (18)	0.0507 (4)	
C11	0.1303 (3)	0.6789 (3)	0.6741 (3)	0.0840 (7)	
H11A	0.1415	0.5884	0.6277	0.126*	
H11B	0.0301	0.6674	0.6704	0.126*	
H11C	0.1944	0.7116	0.7704	0.126*	
C12	0.1746 (3)	1.1619 (2)	0.6375 (2)	0.0740 (6)	
H12A	0.1987	1.2185	0.5799	0.111*	
H12B	0.2405	1.2061	0.7326	0.111*	
H12C	0.0752	1.1559	0.6342	0.111*	
N1	0.4546 (2)	0.6754 (3)	−0.0364 (2)	0.0970 (8)	
N2	0.09232 (17)	0.56946 (15)	0.18014 (14)	0.0511 (4)	
N3	0.18912 (16)	1.01709 (16)	0.58517 (15)	0.0531 (4)	
N4	0.16852 (17)	0.78490 (17)	0.60370 (15)	0.0529 (4)	
O1	0.4045 (2)	0.5528 (3)	−0.1143 (2)	0.1098 (7)	
O2	0.5474 (3)	0.7651 (4)	−0.0482 (3)	0.1804 (16)	
O3	0.0792 (2)	0.44075 (15)	0.13971 (17)	0.0812 (5)	
O4	−0.00550 (14)	0.61913 (14)	0.20145 (14)	0.0609 (4)	
O5	0.22047 (16)	0.61540 (14)	0.45327 (13)	0.0597 (4)	
O6	0.2581 (2)	1.08059 (16)	0.41394 (18)	0.0814 (5)	
O7	0.1253 (2)	0.95645 (19)	0.75936 (16)	0.0872 (5)	
N5	−0.0051 (4)	0.9761 (3)	0.9524 (3)	0.0548 (8)	0.5
H5A	0.0157 (18)	0.9501 (17)	0.8715 (19)	0.066*	0.5
C13	0.0093 (9)	1.1276 (5)	0.9872 (7)	0.0839 (16)	0.5
H13A	−0.062 (5)	1.134 (8)	0.905 (4)	0.126*	0.5
H13B	0.106 (3)	1.180 (6)	0.993 (6)	0.126*	0.5
H13C	−0.010 (6)	1.166 (6)	1.070 (4)	0.126*	0.5
C14	−0.1578 (5)	0.8892 (6)	0.9182 (5)	0.0772 (12)	0.5
H14A	−0.155 (6)	0.791 (3)	0.896 (5)	0.116*	0.5
H14B	−0.217 (6)	0.909 (5)	0.841 (3)	0.116*	0.5
H14C	−0.188 (6)	0.913 (5)	0.998 (4)	0.116*	0.5
C15	0.0966 (8)	0.9352 (8)	1.0574 (6)	0.0886 (17)	0.5
H15A	0.094 (7)	0.834 (2)	1.037 (7)	0.133*	0.5
H15B	0.087 (7)	0.966 (6)	1.148 (4)	0.133*	0.5
H15C	0.193 (4)	0.987 (5)	1.062 (7)	0.133*	0.5
N6	0.4670 (5)	0.5181 (5)	0.4932 (5)	0.0741 (11)	0.5
H6A	0.381 (2)	0.544 (2)	0.4697 (18)	0.089*	0.5
C16	0.4046 (7)	0.3737 (6)	0.3630 (6)	0.0930 (15)	0.5
H16A	0.426 (6)	0.307 (6)	0.413 (6)	0.140*	0.5
H16B	0.441 (6)	0.364 (7)	0.288 (5)	0.140*	0.5
H16C	0.302 (3)	0.367 (7)	0.334 (6)	0.140*	0.5
C17	0.5736 (8)	0.6071 (8)	0.4681 (12)	0.117 (3)	0.5
H17A	0.623 (8)	0.537 (7)	0.436 (7)	0.176*	0.5
H17B	0.647 (7)	0.679 (6)	0.551 (4)	0.176*	0.5
H17C	0.545 (4)	0.651 (6)	0.396 (5)	0.176*	0.5
C18	0.4811 (9)	0.4741 (12)	0.6114 (7)	0.111 (3)	0.5
H18A	0.393 (3)	0.443 (7)	0.631 (5)	0.166*	0.5
H18B	0.558 (5)	0.536 (7)	0.695 (5)	0.166*	0.5
H18C	0.510 (8)	0.389 (5)	0.574 (8)	0.166*	0.5

### Atomic displacement parameters (Å^2^)

**Table:**

	*U*^11^	*U*^22^	*U*^33^	*U*^12^	*U*^13^	*U*^23^
C1	0.0500 (10)	0.1118 (17)	0.0387 (9)	0.0263 (11)	0.0228 (8)	0.0172 (10)
C2	0.0573 (10)	0.0737 (12)	0.0377 (8)	0.0300 (9)	0.0197 (7)	0.0106 (8)
C3	0.0482 (8)	0.0543 (10)	0.0350 (8)	0.0210 (7)	0.0183 (6)	0.0129 (7)
C4	0.0397 (7)	0.0541 (9)	0.0346 (7)	0.0155 (7)	0.0139 (6)	0.0121 (7)
C5	0.0464 (9)	0.0752 (13)	0.0454 (9)	0.0029 (8)	0.0181 (7)	0.0112 (9)
C6	0.0459 (9)	0.1101 (18)	0.0544 (11)	0.0043 (10)	0.0249 (8)	0.0231 (12)
C7	0.0404 (7)	0.0468 (9)	0.0351 (8)	0.0110 (6)	0.0152 (6)	0.0064 (6)
C8	0.0445 (8)	0.0548 (10)	0.0349 (8)	0.0194 (7)	0.0168 (6)	0.0117 (7)
C9	0.0517 (9)	0.0693 (12)	0.0403 (9)	0.0141 (8)	0.0210 (7)	−0.0002 (8)
C10	0.0517 (9)	0.0468 (10)	0.0464 (9)	0.0059 (7)	0.0196 (7)	0.0038 (7)
C11	0.119 (2)	0.0999 (18)	0.0632 (13)	0.0394 (15)	0.0570 (14)	0.0414 (13)
C12	0.0747 (13)	0.0577 (12)	0.0777 (14)	0.0182 (10)	0.0309 (11)	−0.0072 (10)
N1	0.0689 (12)	0.170 (2)	0.0540 (11)	0.0302 (14)	0.0373 (10)	0.0186 (14)
N2	0.0666 (9)	0.0447 (8)	0.0390 (7)	0.0118 (7)	0.0222 (6)	0.0046 (6)
N3	0.0530 (8)	0.0492 (8)	0.0498 (8)	0.0115 (6)	0.0226 (6)	−0.0024 (7)
N4	0.0604 (9)	0.0676 (10)	0.0393 (7)	0.0214 (7)	0.0264 (6)	0.0172 (7)
O1	0.1167 (15)	0.164 (2)	0.0614 (10)	0.0629 (14)	0.0513 (10)	0.0114 (12)
O2	0.145 (2)	0.251 (3)	0.1205 (19)	−0.023 (2)	0.1083 (19)	−0.003 (2)
O3	0.1220 (13)	0.0443 (8)	0.0793 (10)	0.0157 (8)	0.0525 (10)	0.0058 (7)
O4	0.0511 (7)	0.0642 (8)	0.0588 (8)	0.0074 (6)	0.0239 (6)	0.0030 (6)
O5	0.0829 (9)	0.0629 (8)	0.0538 (7)	0.0374 (7)	0.0356 (7)	0.0264 (6)
O6	0.1232 (13)	0.0492 (8)	0.0846 (11)	0.0192 (8)	0.0583 (10)	0.0191 (7)
O7	0.1065 (12)	0.1035 (12)	0.0624 (9)	0.0314 (10)	0.0580 (9)	0.0071 (8)
N5	0.086 (2)	0.0557 (19)	0.0494 (17)	0.0395 (17)	0.0426 (18)	0.0251 (15)
C13	0.126 (5)	0.060 (3)	0.094 (4)	0.042 (3)	0.063 (4)	0.031 (3)
C14	0.082 (3)	0.080 (3)	0.064 (3)	0.016 (2)	0.030 (2)	0.010 (2)
C15	0.100 (4)	0.117 (5)	0.077 (3)	0.063 (4)	0.032 (3)	0.050 (4)
N6	0.081 (3)	0.082 (3)	0.104 (3)	0.048 (2)	0.059 (3)	0.055 (2)
C16	0.106 (4)	0.081 (4)	0.090 (4)	0.026 (3)	0.039 (3)	0.016 (3)
C17	0.087 (4)	0.076 (4)	0.195 (9)	0.017 (3)	0.042 (5)	0.065 (5)
C18	0.106 (5)	0.192 (9)	0.083 (4)	0.090 (6)	0.054 (4)	0.067 (5)

### Geometric parameters (Å, º)

**Table:**

C1—C2	1.358 (3)	C13—C14^i^	1.577 (10)
C1—C6	1.377 (3)	C13—H13A	0.962 (19)
C1—N1	1.471 (2)	C13—H13B	0.971 (19)
C2—C3	1.385 (2)	C13—H13C	0.944 (18)
C2—H2	0.9300	C14—C13^i^	1.577 (10)
C3—C4	1.403 (2)	C14—C15^i^	1.669 (9)
C3—N2	1.471 (2)	C14—N5^i^	1.819 (6)
C4—C5	1.400 (2)	C14—H14A	0.962 (18)
C4—C7	1.463 (2)	C14—H14B	0.938 (18)
C5—C6	1.379 (3)	C14—H14C	0.956 (18)
C5—H5	0.9300	C15—C13^i^	1.019 (8)
C6—H6	0.9300	C15—N5^i^	1.375 (6)
C7—C8	1.403 (2)	C15—C14^i^	1.669 (9)
C7—C10	1.411 (2)	C15—H15A	0.972 (19)
C8—O5	1.243 (2)	C15—H15B	0.960 (19)
C8—N4	1.404 (2)	C15—H15C	0.973 (19)
C9—O7	1.219 (2)	N6—N6^ii^	0.796 (6)
C9—N3	1.365 (3)	N6—C18^ii^	1.340 (8)
C9—N4	1.370 (2)	N6—C17	1.367 (8)
C10—O6	1.231 (2)	N6—C18	1.381 (7)
C10—N3	1.408 (2)	N6—C17^ii^	1.419 (8)
C11—N4	1.470 (3)	N6—C16	1.603 (7)
C11—H11A	0.9600	N6—C16^ii^	1.632 (8)
C11—H11B	0.9600	N6—H6A	0.92 (2)
C11—H11C	0.9600	C16—C18^ii^	1.621 (12)
C12—N3	1.466 (2)	C16—N6^ii^	1.633 (8)
C12—H12A	0.9600	C16—C17^ii^	1.673 (13)
C12—H12B	0.9600	C16—H16A	0.955 (19)
C12—H12C	0.9600	C16—H16B	0.938 (19)
N1—O2	1.206 (3)	C16—H16C	0.953 (19)
N1—O1	1.215 (3)	C17—C18^ii^	0.949 (9)
N2—O4	1.2122 (19)	C17—N6^ii^	1.419 (8)
N2—O3	1.2205 (19)	C17—C16^ii^	1.673 (13)
N5—N5^i^	0.964 (5)	C17—H17A	0.98 (2)
N5—C13^i^	1.328 (6)	C17—H17B	0.97 (2)
N5—C15^i^	1.375 (6)	C17—H17C	0.955 (18)
N5—C13	1.435 (6)	C18—C17^ii^	0.949 (9)
N5—C15	1.450 (6)	C18—N6^ii^	1.340 (8)
N5—C14	1.482 (6)	C18—C16^ii^	1.621 (12)
N5—C14^i^	1.819 (6)	C18—H18A	0.957 (18)
N5—H5A	0.914 (19)	C18—H18B	0.956 (19)
C13—C15^i^	1.019 (8)	C18—H18C	0.971 (19)
C13—N5^i^	1.328 (6)		
			
C2—C1—C6	121.79 (16)	N5—C14—H14C	110 (4)
C2—C1—N1	117.9 (2)	C13^i^—C14—H14C	90 (3)
C6—C1—N1	120.3 (2)	C15^i^—C14—H14C	89 (3)
C1—C2—C3	117.72 (17)	N5^i^—C14—H14C	78 (3)
C1—C2—H2	121.1	H14A—C14—H14C	109 (3)
C3—C2—H2	121.1	H14B—C14—H14C	113 (3)
C2—C3—C4	123.94 (16)	C13^i^—C15—N5^i^	72.0 (5)
C2—C3—N2	114.20 (15)	C13^i^—C15—N5	62.2 (4)
C4—C3—N2	121.62 (13)	N5^i^—C15—N5	39.8 (2)
C5—C4—C3	114.84 (14)	C13^i^—C15—C14^i^	127.8 (6)
C5—C4—C7	121.04 (15)	N5^i^—C15—C14^i^	57.3 (3)
C3—C4—C7	124.08 (14)	N5—C15—C14^i^	70.9 (4)
C6—C5—C4	122.27 (18)	C13^i^—C15—H15A	69 (4)
C6—C5—H5	118.9	N5^i^—C15—H15A	141 (4)
C4—C5—H5	118.9	N5—C15—H15A	117 (5)
C5—C6—C1	119.30 (18)	C14^i^—C15—H15A	159 (3)
C5—C6—H6	120.3	C13^i^—C15—H15B	93 (4)
C1—C6—H6	120.3	N5^i^—C15—H15B	73 (4)
C8—C7—C10	121.30 (14)	N5—C15—H15B	112 (4)
C8—C7—C4	119.04 (14)	C14^i^—C15—H15B	84 (4)
C10—C7—C4	119.64 (15)	H15A—C15—H15B	108 (3)
O5—C8—N4	117.22 (15)	C13^i^—C15—H15C	158 (4)
O5—C8—C7	125.47 (14)	N5^i^—C15—H15C	109 (4)
N4—C8—C7	117.30 (15)	N5—C15—H15C	104 (4)
O7—C9—N3	121.10 (19)	C14^i^—C15—H15C	53 (4)
O7—C9—N4	121.4 (2)	H15A—C15—H15C	107 (3)
N3—C9—N4	117.52 (14)	H15B—C15—H15C	109 (3)
O6—C10—N3	117.41 (16)	N6^ii^—N6—C18^ii^	75.8 (6)
O6—C10—C7	125.85 (16)	N6^ii^—N6—C17	77.1 (7)
N3—C10—C7	116.72 (16)	C18^ii^—N6—C17	41.0 (4)
N4—C11—H11A	109.5	N6^ii^—N6—C18	70.2 (7)
N4—C11—H11B	109.5	C18^ii^—N6—C18	146.0 (3)
H11A—C11—H11B	109.5	C17—N6—C18	127.1 (6)
N4—C11—H11C	109.5	N6^ii^—N6—C17^ii^	69.8 (7)
H11A—C11—H11C	109.5	C18^ii^—N6—C17^ii^	126.1 (6)
H11B—C11—H11C	109.5	C17—N6—C17^ii^	146.9 (3)
N3—C12—H12A	109.5	C18—N6—C17^ii^	39.6 (4)
N3—C12—H12B	109.5	N6^ii^—N6—C16	77.8 (7)
H12A—C12—H12B	109.5	C18^ii^—N6—C16	66.1 (5)
N3—C12—H12C	109.5	C17—N6—C16	106.5 (6)
H12A—C12—H12C	109.5	C18—N6—C16	105.7 (6)
H12B—C12—H12C	109.5	C17^ii^—N6—C16	66.9 (6)
O2—N1—O1	124.0 (2)	N6^ii^—N6—C16^ii^	73.7 (8)
O2—N1—C1	116.9 (3)	C18^ii^—N6—C16^ii^	106.1 (5)
O1—N1—C1	119.1 (2)	C17—N6—C16^ii^	67.1 (6)
O4—N2—O3	123.40 (16)	C18—N6—C16^ii^	64.5 (5)
O4—N2—C3	118.26 (14)	C17^ii^—N6—C16^ii^	102.6 (6)
O3—N2—C3	118.14 (15)	C16—N6—C16^ii^	151.5 (2)
C9—N3—C10	123.73 (15)	N6^ii^—N6—H6A	170.3 (15)
C9—N3—C12	117.55 (16)	C18^ii^—N6—H6A	104.8 (11)
C10—N3—C12	118.73 (17)	C17—N6—H6A	109.9 (11)
C9—N4—C8	123.40 (15)	C18—N6—H6A	108.7 (11)
C9—N4—C11	118.16 (15)	C17^ii^—N6—H6A	103.0 (11)
C8—N4—C11	118.44 (17)	C16—N6—H6A	93.5 (12)
N5^i^—N5—C13^i^	75.7 (4)	C16^ii^—N6—H6A	114.8 (11)
N5^i^—N5—C15^i^	74.3 (4)	N6—C16—C18^ii^	49.1 (3)
C13^i^—N5—C15^i^	127.0 (4)	N6—C16—N6^ii^	28.5 (2)
N5^i^—N5—C13	63.7 (4)	C18^ii^—C16—N6^ii^	50.2 (3)
C13^i^—N5—C13	139.4 (3)	N6—C16—C17^ii^	51.3 (3)
C15^i^—N5—C13	42.4 (3)	C18^ii^—C16—C17^ii^	96.6 (4)
N5^i^—N5—C15	65.9 (4)	N6^ii^—C16—C17^ii^	48.8 (3)
C13^i^—N5—C15	42.7 (4)	N6—C16—H16A	99 (4)
C15^i^—N5—C15	140.2 (2)	C18^ii^—C16—H16A	125 (4)
C13—N5—C15	113.9 (4)	N6^ii^—C16—H16A	80 (4)
N5^i^—N5—C14	93.7 (4)	C17^ii^—C16—H16A	53 (4)
C13^i^—N5—C14	68.1 (4)	N6—C16—H16B	121 (4)
C15^i^—N5—C14	71.4 (4)	C18^ii^—C16—H16B	72 (4)
C13—N5—C14	112.9 (4)	N6^ii^—C16—H16B	110 (4)
C15—N5—C14	110.2 (4)	C17^ii^—C16—H16B	152 (4)
N5^i^—N5—C14^i^	54.4 (4)	H16A—C16—H16B	112 (3)
C13^i^—N5—C14^i^	100.0 (4)	N6—C16—H16C	101 (4)
C15^i^—N5—C14^i^	96.7 (4)	C18^ii^—C16—H16C	119 (4)
C13—N5—C14^i^	56.5 (4)	N6^ii^—C16—H16C	127 (4)
C15—N5—C14^i^	60.2 (4)	C17^ii^—C16—H16C	96 (4)
C14—N5—C14^i^	148.1 (2)	H16A—C16—H16C	110 (3)
N5^i^—N5—H5A	161.2 (12)	H16B—C16—H16C	113 (3)
C13^i^—N5—H5A	111.6 (10)	C18^ii^—C17—N6	68.0 (6)
C15^i^—N5—H5A	110.8 (10)	C18^ii^—C17—N6^ii^	68.0 (6)
C13—N5—H5A	107.2 (11)	N6—C17—N6^ii^	33.1 (3)
C15—N5—H5A	107.0 (11)	C18^ii^—C17—C16^ii^	128.5 (9)
C14—N5—H5A	105.1 (11)	N6—C17—C16^ii^	64.0 (5)
C14^i^—N5—H5A	106.8 (11)	N6^ii^—C17—C16^ii^	61.8 (5)
C15^i^—C13—N5^i^	75.1 (5)	C18^ii^—C17—H17A	61 (5)
C15^i^—C13—N5	65.6 (5)	N6—C17—H17A	99 (5)
N5^i^—C13—N5	40.6 (3)	N6^ii^—C17—H17A	70 (5)
C15^i^—C13—C14^i^	134.6 (6)	C16^ii^—C17—H17A	109 (4)
N5^i^—C13—C14^i^	60.6 (4)	C18^ii^—C17—H17B	167 (4)
N5—C13—C14^i^	74.1 (4)	N6—C17—H17B	115 (5)
C15^i^—C13—H13A	56 (4)	N6^ii^—C17—H17B	107 (4)
N5^i^—C13—H13A	129 (4)	C16^ii^—C17—H17B	51 (5)
N5—C13—H13A	102 (5)	H17A—C17—H17B	106 (3)
C14^i^—C13—H13A	159 (4)	C18^ii^—C17—H17C	78 (4)
C15^i^—C13—H13B	158 (3)	N6—C17—H17C	119.0 (18)
N5^i^—C13—H13B	117 (4)	N6^ii^—C17—H17C	143 (3)
N5—C13—H13B	111 (4)	C16^ii^—C17—H17C	142 (4)
C14^i^—C13—H13B	57 (4)	H17A—C17—H17C	108 (3)
H13A—C13—H13B	107 (3)	H17B—C17—H17C	109 (3)
C15^i^—C13—H13C	89 (3)	C17^ii^—C18—N6^ii^	71.0 (7)
N5^i^—C13—H13C	76 (4)	C17^ii^—C18—N6	72.4 (7)
N5—C13—H13C	114 (4)	N6^ii^—C18—N6	34.0 (3)
C14^i^—C13—H13C	89 (4)	C17^ii^—C18—C16^ii^	134.5 (9)
H13A—C13—H13C	112 (3)	N6^ii^—C18—C16^ii^	64.8 (5)
H13B—C13—H13C	111 (3)	N6—C18—C16^ii^	65.3 (5)
N5—C14—C13^i^	51.3 (3)	C17^ii^—C18—H18A	78 (4)
N5—C14—C15^i^	51.3 (3)	N6^ii^—C18—H18A	143 (3)
C13^i^—C14—C15^i^	96.3 (3)	N6—C18—H18A	117.4 (17)
N5—C14—N5^i^	31.9 (2)	C16^ii^—C18—H18A	136 (4)
C13^i^—C14—N5^i^	49.4 (3)	C17^ii^—C18—H18B	161 (5)
C15^i^—C14—N5^i^	48.9 (3)	N6^ii^—C18—H18B	105 (4)
N5—C14—H14A	106 (4)	N6—C18—H18B	116 (5)
C13^i^—C14—H14A	69 (3)	C16^ii^—C18—H18B	50 (5)
C15^i^—C14—H14A	156 (3)	H18A—C18—H18B	110 (3)
N5^i^—C14—H14A	118 (3)	C17^ii^—C18—H18C	51 (5)
N5—C14—H14B	107 (4)	N6^ii^—C18—H18C	67 (5)
C13^i^—C14—H14B	154 (4)	N6—C18—H18C	94 (5)
C15^i^—C14—H14B	73 (3)	C16^ii^—C18—H18C	116 (4)
N5^i^—C14—H14B	122 (4)	H18A—C18—H18C	108 (3)
H14A—C14—H14B	112 (3)	H18B—C18—H18C	110 (3)
			
C6—C1—C2—C3	0.4 (3)	C13^i^—N5—C14—C15^i^	145.1 (4)
N1—C1—C2—C3	−179.46 (18)	C13—N5—C14—C15^i^	9.2 (4)
C1—C2—C3—C4	−3.7 (3)	C15—N5—C14—C15^i^	137.8 (3)
C1—C2—C3—N2	170.69 (17)	C14^i^—N5—C14—C15^i^	72.1 (4)
C2—C3—C4—C5	4.0 (3)	C13^i^—N5—C14—N5^i^	73.0 (4)
N2—C3—C4—C5	−169.94 (16)	C15^i^—N5—C14—N5^i^	−72.1 (4)
C2—C3—C4—C7	−173.67 (16)	C13—N5—C14—N5^i^	−62.9 (4)
N2—C3—C4—C7	12.4 (2)	C15—N5—C14—N5^i^	65.6 (4)
C3—C4—C5—C6	−1.2 (3)	C14^i^—N5—C14—N5^i^	−0.002 (2)
C7—C4—C5—C6	176.58 (18)	N5^i^—N5—C15—C13^i^	95.0 (6)
C4—C5—C6—C1	−1.8 (3)	C15^i^—N5—C15—C13^i^	95.0 (6)
C2—C1—C6—C5	2.3 (3)	C13—N5—C15—C13^i^	138.1 (4)
N1—C1—C6—C5	−177.9 (2)	C14—N5—C15—C13^i^	10.1 (6)
C5—C4—C7—C8	−136.83 (18)	C14^i^—N5—C15—C13^i^	156.4 (6)
C3—C4—C7—C8	40.7 (2)	C13^i^—N5—C15—N5^i^	−95.0 (6)
C5—C4—C7—C10	41.5 (2)	C15^i^—N5—C15—N5^i^	0.000 (1)
C3—C4—C7—C10	−140.90 (17)	C13—N5—C15—N5^i^	43.1 (4)
C10—C7—C8—O5	−178.11 (16)	C14—N5—C15—N5^i^	−84.9 (5)
C4—C7—C8—O5	0.2 (2)	C14^i^—N5—C15—N5^i^	61.4 (4)
C10—C7—C8—N4	0.7 (2)	N5^i^—N5—C15—C14^i^	−61.4 (4)
C4—C7—C8—N4	179.05 (14)	C13^i^—N5—C15—C14^i^	−156.4 (6)
C8—C7—C10—O6	−179.24 (18)	C15^i^—N5—C15—C14^i^	−61.4 (4)
C4—C7—C10—O6	2.4 (3)	C13—N5—C15—C14^i^	−18.3 (4)
C8—C7—C10—N3	−0.9 (2)	C14—N5—C15—C14^i^	−146.3 (2)
C4—C7—C10—N3	−179.29 (14)	N6^ii^—N6—C16—C18^ii^	−79.7 (6)
C2—C1—N1—O2	174.1 (3)	C17—N6—C16—C18^ii^	−7.4 (6)
C6—C1—N1—O2	−5.7 (4)	C18—N6—C16—C18^ii^	−144.9 (3)
C2—C1—N1—O1	−4.5 (3)	C17^ii^—N6—C16—C18^ii^	−152.8 (6)
C6—C1—N1—O1	175.7 (2)	C16^ii^—N6—C16—C18^ii^	−79.7 (6)
C2—C3—N2—O4	−135.67 (17)	C18^ii^—N6—C16—N6^ii^	79.7 (6)
C4—C3—N2—O4	38.8 (2)	C17—N6—C16—N6^ii^	72.4 (7)
C2—C3—N2—O3	39.5 (2)	C18—N6—C16—N6^ii^	−65.1 (6)
C4—C3—N2—O3	−146.01 (17)	C17^ii^—N6—C16—N6^ii^	−73.0 (7)
O7—C9—N3—C10	177.49 (17)	C16^ii^—N6—C16—N6^ii^	0.004 (2)
N4—C9—N3—C10	−2.1 (3)	N6^ii^—N6—C16—C17^ii^	73.0 (7)
O7—C9—N3—C12	−2.2 (3)	C18^ii^—N6—C16—C17^ii^	152.8 (6)
N4—C9—N3—C12	178.26 (16)	C17—N6—C16—C17^ii^	145.4 (4)
O6—C10—N3—C9	−179.88 (18)	C18—N6—C16—C17^ii^	7.9 (6)
C7—C10—N3—C9	1.7 (2)	C16^ii^—N6—C16—C17^ii^	73.0 (7)
O6—C10—N3—C12	−0.2 (3)	N6^ii^—N6—C17—C18^ii^	83.2 (10)
C7—C10—N3—C12	−178.68 (16)	C18—N6—C17—C18^ii^	135.7 (5)
O7—C9—N4—C8	−177.78 (17)	C17^ii^—N6—C17—C18^ii^	83.2 (10)
N3—C9—N4—C8	1.8 (3)	C16—N6—C17—C18^ii^	10.3 (8)
O7—C9—N4—C11	2.9 (3)	C16^ii^—N6—C17—C18^ii^	160.8 (9)
N3—C9—N4—C11	−177.50 (18)	C18^ii^—N6—C17—N6^ii^	−83.2 (10)
O5—C8—N4—C9	177.77 (15)	C18—N6—C17—N6^ii^	52.5 (7)
C7—C8—N4—C9	−1.1 (2)	C17^ii^—N6—C17—N6^ii^	−0.001 (2)
O5—C8—N4—C11	−2.9 (2)	C16—N6—C17—N6^ii^	−72.9 (7)
C7—C8—N4—C11	178.16 (17)	C16^ii^—N6—C17—N6^ii^	77.6 (8)
N5^i^—N5—C13—C15^i^	−95.4 (6)	N6^ii^—N6—C17—C16^ii^	−77.6 (8)
C13^i^—N5—C13—C15^i^	−95.4 (6)	C18^ii^—N6—C17—C16^ii^	−160.8 (9)
C15—N5—C13—C15^i^	−139.6 (4)	C18—N6—C17—C16^ii^	−25.1 (7)
C14—N5—C13—C15^i^	−13.0 (6)	C17^ii^—N6—C17—C16^ii^	−77.6 (8)
C14^i^—N5—C13—C15^i^	−158.6 (6)	C16—N6—C17—C16^ii^	−150.5 (3)
C13^i^—N5—C13—N5^i^	0.000 (1)	N6^ii^—N6—C18—C17^ii^	−81.9 (10)
C15^i^—N5—C13—N5^i^	95.4 (6)	C18^ii^—N6—C18—C17^ii^	−81.9 (10)
C15—N5—C13—N5^i^	−44.1 (4)	C17—N6—C18—C17^ii^	−137.2 (5)
C14—N5—C13—N5^i^	82.5 (5)	C16—N6—C18—C17^ii^	−11.5 (8)
C14^i^—N5—C13—N5^i^	−63.1 (4)	C16^ii^—N6—C18—C17^ii^	−162.9 (8)
N5^i^—N5—C13—C14^i^	63.1 (4)	C18^ii^—N6—C18—N6^ii^	−0.002 (2)
C13^i^—N5—C13—C14^i^	63.1 (4)	C17—N6—C18—N6^ii^	−55.2 (8)
C15^i^—N5—C13—C14^i^	158.6 (6)	C17^ii^—N6—C18—N6^ii^	81.9 (10)
C15—N5—C13—C14^i^	19.0 (4)	C16—N6—C18—N6^ii^	70.4 (7)
C14—N5—C13—C14^i^	145.6 (3)	C16^ii^—N6—C18—N6^ii^	−80.9 (7)
N5^i^—N5—C14—C13^i^	−73.0 (4)	N6^ii^—N6—C18—C16^ii^	80.9 (7)
C15^i^—N5—C14—C13^i^	−145.1 (4)	C18^ii^—N6—C18—C16^ii^	80.9 (7)
C13—N5—C14—C13^i^	−135.9 (3)	C17—N6—C18—C16^ii^	25.7 (7)
C15—N5—C14—C13^i^	−7.4 (4)	C17^ii^—N6—C18—C16^ii^	162.9 (8)
C14^i^—N5—C14—C13^i^	−73.0 (4)	C16—N6—C18—C16^ii^	151.4 (3)
N5^i^—N5—C14—C15^i^	72.1 (4)		

Symmetry codes: (i) −*x*, −*y*+2, −*z*+2; (ii) −*x*+1, −*y*+1, −*z*+1.

### Hydrogen-bond geometry (Å, º)

**Table:**

*D*—H···*A*	*D*—H	H···*A*	*D*···*A*	*D*—H···*A*
N5—H5*A*···O7	0.91 (2)	1.80 (2)	2.666 (3)	156 (2)
N6—H6*A*···O5	0.92 (2)	1.83 (2)	2.737 (3)	171 (2)
