# Supplementary figures and images for: Crystal structure of tri­methyl­ammonium 5-(2,4-di­nitro­phen­yl)-1,3-dimethyl-2,6-dioxo-1,2,3,6-tetra­hydro­pyrimidin-4-olate
Source: Acta Crystallogr Sect E Struct Rep Online. 2014 Sep 13;70(Pt 10):o1102–3. doi: 10.1107/S1600536814019977 (PMC4257181; doi:10.1107/S1600536814019977)

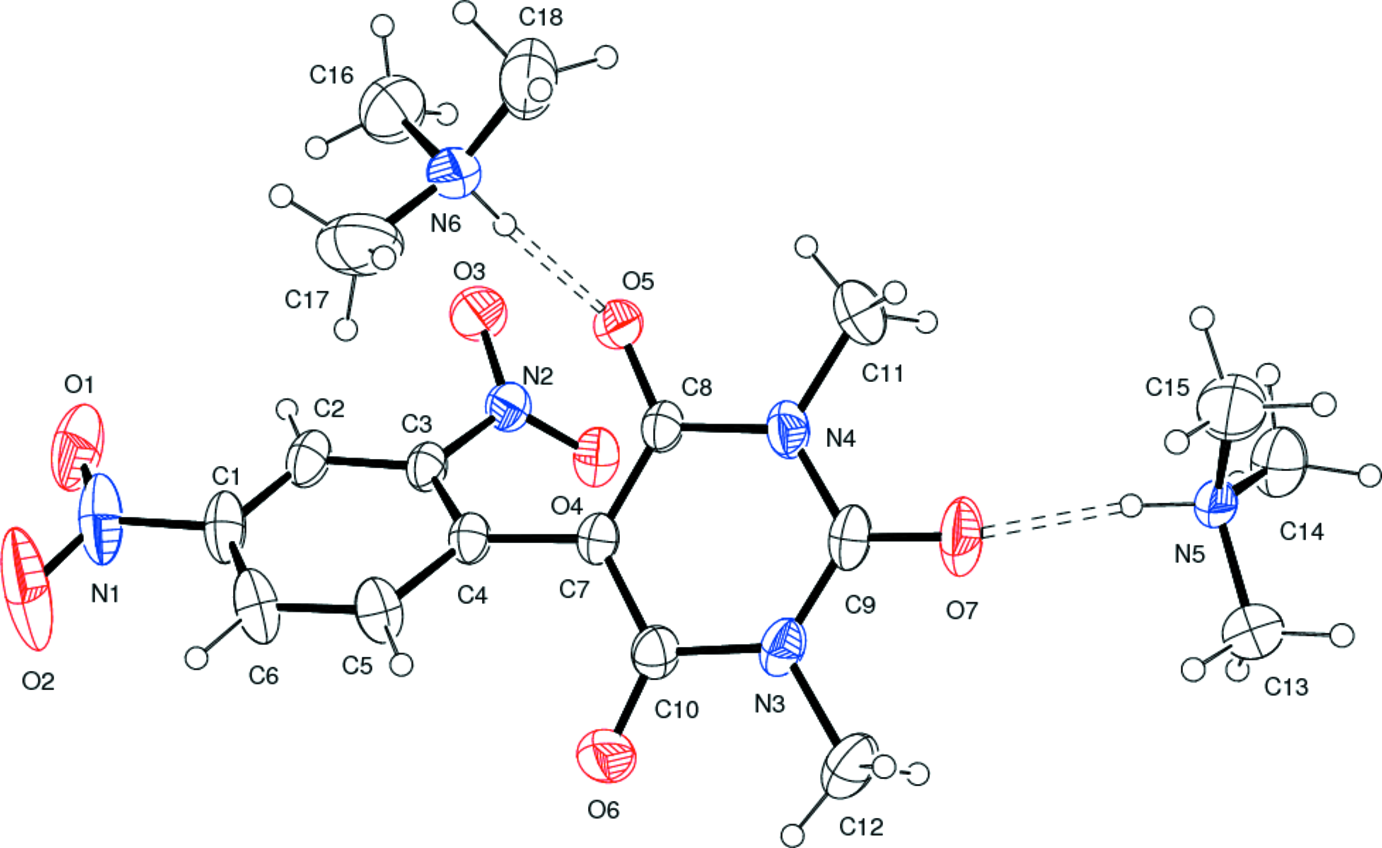

Supplement: Supplementary file 4 [file e-70-o1102-fig1.tif]
